# Direct stacking of sequence-specific nuclease-induced mutations to produce high oleic and low linolenic soybean oil

**DOI:** 10.1186/s12870-016-0906-1

**Published:** 2016-10-13

**Authors:** Zachary L. Demorest, Andrew Coffman, Nicholas J. Baltes, Thomas J. Stoddard, Benjamin M. Clasen, Song Luo, Adam Retterath, Ann Yabandith, Maria Elena Gamo, Jeff Bissen, Luc Mathis, Daniel F. Voytas, Feng Zhang

**Affiliations:** Calyxt, Inc., 600 County Road D West Suite 8, New Brighton, MN 55112 USA

**Keywords:** TALEN, Genome editing, Soybean, High oleic acid, Low linolenic acid, Soybean oil, Targeted mutagenesis

## Abstract

**Background:**

The ability to modulate levels of individual fatty acids within soybean oil has potential to increase shelf-life and frying stability and to improve nutritional characteristics. Commodity soybean oil contains high levels of polyunsaturated linoleic and linolenic acid, which contribute to oxidative instability – a problem that has been addressed through partial hydrogenation. However, partial hydrogenation increases levels of *trans*-fatty acids, which have been associated with cardiovascular disease. Previously, we generated soybean lines with knockout mutations within fatty acid desaturase 2-1A (*FAD2-1A*) and *FAD2-1B* genes, resulting in oil with increased levels of monounsaturated oleic acid (18:1) and decreased levels of linoleic (18:2) and linolenic acid (18:3). Here, we stack mutations within *FAD2-1A* and *FAD2-1B* with mutations in fatty acid desaturase 3A (*FAD3A*) to further decrease levels of linolenic acid. Mutations were introduced into *FAD3A* by directly delivering TALENs into *fad2-1a fad2-1b* soybean plants.

**Results:**

Oil from *fad2-1a fad2-1b fad3a* plants had significantly lower levels of linolenic acid (2.5 %), as compared to *fad2-1a fad2-1b* plants (4.7 %). Furthermore, oil had significantly lower levels of linoleic acid (2.7 % compared to 5.1 %) and significantly higher levels of oleic acid (82.2 % compared to 77.5 %). Transgene-free *fad2-1a fad2-1b fad3a* soybean lines were identified.

**Conclusions:**

The methods presented here provide an efficient means for using sequence-specific nucleases to stack quality traits in soybean. The resulting product comprised oleic acid levels above 80 % and linoleic and linolenic acid levels below 3 %.

**Electronic supplementary material:**

The online version of this article (doi:10.1186/s12870-016-0906-1) contains supplementary material, which is available to authorized users.

## Background

Soybean is an important legume crop that is valued for both its protein and oil content. Worldwide soybean production in 2014/2015 was 319 million metric tons, with 108 million metric tons produced in the United States. Soybean oil is used in applications ranging from cooking and frying to industrial lubrication and biofuels. Commodity soybean oil is primarily composed of five fatty acids: palmitic acid (~13 %, saturated, 16:0), stearic acid (~4 %, saturated, 18:0), oleic acid (~20 %, monounsaturated, 18:1), linoleic acid (~55 %, polyunsaturated, 18:2) and linolenic acid (~8 %, polyunsaturated, 18:3). Due to high levels of polyunsaturated fatty acids, soybean oil has poor oxidative and frying stability, which limits its use in food products and industrial applications. In an effort to lower the levels of polyunsaturated fatty acids, soybean oil is partially hydrogenated; however, partial hydrogenation significantly increases *trans*-fatty acids, which have been linked with coronary heart disease and buildup of plaque in arteries [1]. The Food and Drug Administration (FDA) made a preliminary determination that partially hydrogenated oils are no longer ‘generally recognized as safe’ (GRAS) and is now taking steps to remove artificial *trans* fats from human food [2]. Altering the composition of soybean oil by decreasing the levels of polyunsaturated fatty acids may help reduce the need for hydrogenation.

Significant progress has been made in uncovering the genes involved in the soybean lipid biosynthetic pathway, and those involved in conversion of oleic acid into polyunsaturated fatty acids. Conversion of oleic to linoleic acid is catalyzed by fatty acid desaturase 2 (FAD2) proteins [3]. There are three *FAD2* desaturase genes within the soybean genome, *FAD2-1A* (Glyma10g42470), *FAD2-1B* (Glyma20g24530) and *FAD2-2* (Glyma03g30070)*.* Both *FAD2-1A* and *FAD2-1B* are highly expressed during peak oil synthesis and are the primary genetic determinants of oleic and linoleic acid levels in soybean seeds [4, 5]. Disruption or decreased expression of *FAD2-1* genes results in oil with elevated oleic acid and decreased linoleic and linolenic acid [6–12]. Combination of mutations within *FAD2-1A* and *FAD2-1B* genes results in soybean oil with oleic acid levels ~ 80 % and linoleic and linolenic acid levels ~ 5 % each [13, 14].

Decreasing levels of linolenic acid is predicted to improve soybean oil characteristics by decreasing total levels of polyunsaturated fatty acids, and subsequently increasing frying and oxidative stability. Conversion of linoleic to linolenic acid is catalyzed by the fatty acid desaturase 3 (FAD3) enzyme, which is produced by a family of genes consisting of *FAD3A* (Glyma14g37350), *FAD3B* (Glyma02g39230) and *FAD3C* (Glyma18g06950). Consistent with its high expression in developing seeds, *FAD3A* has the greatest effect on linolenic acid concentrations in soybean oil [15]. Combining mutations within *FAD3A* with *FAD3B* and/or *FAD3C* resulted in oil having <3 % linolenic acid [16–20]

With the advent of sequence-specific nucleases, including TALENs and CRISPR/Cas, it has become possible to introduce targeted knockout mutations within genes of interest [21]. When delivered to plant cells, sequence-specific nucleases generate targeted DNA double-strand breaks. These double-strand breaks are then repaired predominantly by non-homologous end joining (NHEJ), which may result in the introduction of small insertions or deletions at the repair site. If double-strand breaks are generated within gene coding sequences, imprecise repair by NHEJ has potential to introduce frameshift mutations or in-frame deletions that destroy protein function. The objective of this study was to create high oleic and low linolenic soybean lines by stacking targeted mutations within *FAD2-1A*, *FAD2-1B* and *FAD3* genes. With the current industrial standard for low linolenic acid soybean oil being 3 % [20], we sought to inactivate a sufficient number of *FAD3* genes to achieve linolenic levels <3 %.

## Results

### Designing TALENs targeting the soybean *FAD3* genes

**Fig. 1:**
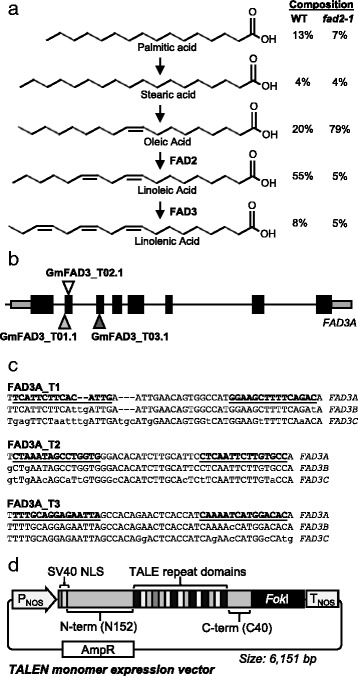
Design of TALENs targeting *FAD3* genes within *Glycine max*. **a** Illustration of the fatty acid pathway. Relative percent composition of individual fatty acids in the oil from WT and *fad2-1* knockout plants is shown on the right. **b** Schematic of the *FAD3A* genomic sequence. Triangles, approximate TALEN binding sites; *black boxes*, exons; *gray boxes*, 5’ and 3’ untranslated regions. **c** Nucleotide sequences of the predicted TALEN target sites within the *FAD3A*, *FAD3B*, and *FAD3A* genes. Bold and underlined nucleotides indicate TALEN binding sequence. Lower case nucleotides indicate positions of SNPs. **d** Illustration of a TALEN monomer expression vector. P_NOS_, nopaline synthase promoter; SV40 NLS, simian virus 40 large T-antigen nuclear localization signal; T_NOS_, nopaline synthase terminator; AmpR, ampicillin resistance gene

Soybean oil is primarily composed of palmitic acid, stearic acid, oleic acid, linoleic acid and linolenic acid. In oil from wild type plants, these five fatty acids are present at approximately 13, 4, 20, 55 and 8 %, respectively (Fig. 1a). Previously, we used TALENs to generate knockout mutations within both *FAD2-1A* and *FAD2-1B* genes [14]. Oil from the resulting plants contained higher levels of oleic acid (~79 %) and lower levels of linoleic and linolenic acids (~5 % each), compared to oil from WT plants. Here, we sought to further improve oil characteristics by knocking out genes involved in the conversion of linoleic to linolenic acid. We predicted that by knocking out the *FAD3* linoleate desaturase genes, levels of linolenic acid would further decrease.

The soybean genome contains three linoleate desaturase genes: *FAD3A* (Glyma14g37350), *FAD3B* (Glyma02g39230) and *FAD3C* (Glyma18g06950). In terms of nucleotide similarity of their coding sequences, *FAD3A* shares 96.2 % identity to *FAD3B* and 14.4 % identity to *FAD3C*. Compared to *FAD3B* and *FAD3C*, mutations in *FAD3A* confer the greatest decrease in linolenic acid levels in soybean oil (from ~8 to ~4 %) [22], which is consistent with higher expression of *FAD3A* within developing seeds [15]. Therefore, we sought to design TALENs that primarily recognize *FAD3A* sequence. Three TALEN pairs were synthesized which recognize sequence within exon two or exon three of *FAD3A* (designated as GmFAD3_T01.1, GmFAD3_T02.1, and GmFAD3_T03.1) (Fig. 1b). TALENs were designed to recognize *FAD3A* sequence which is partially conserved between *FAD3B* and *FAD3C*; however, the recognition sequences for all TALEN pairs at *FAD3B* and *FAD3C* contained at least one single-nucleotide polymorphism (SNP), but up to 11 SNPs, when compared to the *FAD3A* sequence (Fig. 1c).

### Assessing TALEN activity in protoplasts by deep-sequencing

**Fig. 2:**
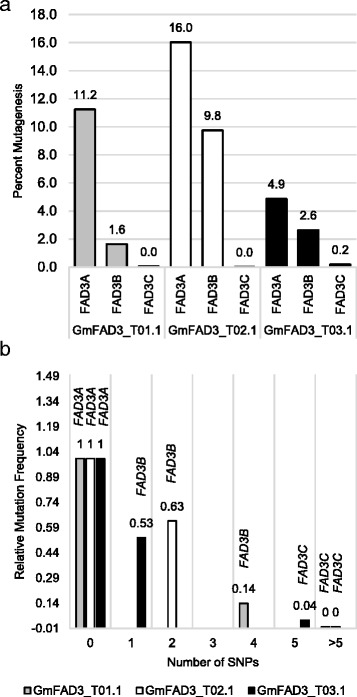
*FAD3* TALEN activity in soybean protoplasts. **a** TALEN pairs were assessed for their activity ~48 h after transformation in soybean protoplast. The frequency of mutagenesis represents the total number of sequence reads with insertions or deletions divided by the total number of sequence reads. The resulting number was then divided by the transformation frequency (90 %) which was determined using a YFP control plasmid. **b** TALEN activity relative to the number of SNPs present within the predicted TALEN binding sites

To determine TALEN activity, protoplasts were transformed with plasmid DNA encoding each TALEN pair and the *FAD3* target sites were deep-sequenced. To this end, approximately 500 000 protoplasts were transformed with 15 μg each of two plasmids encoding a complete TALEN pair. Protoplasts were transformed using polyethylene glycol. Genomic DNA was isolated ~48 h post transformation and used as a template in a PCR with primers designed to individually amplify TALEN target sites within the *FAD3A*, *FAD3B* or *FAD3C* gene. Amplicon pools for each TALEN target site were sequenced by 454 pyrosequencing. For all three TALEN pairs, we observed evidence of NHEJ mutations in two of the three *FAD3* genes (Fig. 2a). TALEN pair GmFAD3_T02.1 introduced mutations within both *FAD3A* and *FAD3B*, and, relative to the other TALEN pairs, had the highest activity at its intended target sequence, *FAD3A* (16.0 %). On the other hand, TALEN pair GmFAD3_T03.1 had the lowest activity at its intended *FAD3A* target sequence (4.9 %). Activity of all three TALEN pairs at the *FAD3B* and *FAD3C* target sites was lower than the respective *FAD3A* target site, which is most likely due to SNPs within the *FAD3B* and *FAD3C* TALEN binding sites.

We observed a correlation between the number of SNPs within TALEN binding sites and the relative mutation frequencies (Fig. 2b). Mutation frequencies at *FAD3A* target sites (containing 0 SNPs) for TALEN pairs GmFAD3_T01.1, GmFAD3_T02.1, GmFAD3_T03.1 were 11.2, 16.0 and 4.9 % respectively. After normalizing TALEN mutation frequencies at *FAD3A*, the relative mutation frequencies at *FAD3B* and *FAD3C* were determined. Target sites with one or two SNPs decreased mutation frequencies to ~53 or 63 %, respectively, relative to the activity of the corresponding TALEN *FAD3A*; target sites with four SNPs decreased mutation frequencies to 14 %; target sites with five SNPs decreased mutation frequencies to 0.041 %, and target sites with >5 SNPs decreased mutation frequencies to undetectable levels. Whereas these data do not account for relative position of the SNPs, they provide evidence for TALEN target site specificity, indicating that target sites with five or more SNPs are unlikely to be recognized and cleaved.

### Generating soybean plants with *FAD3* mutations

To generate soybean plants with knockout mutations in *FAD3* genes, DNA encoding TALEN pair GmFAD3_T02.1 was stably integrated into the soybean genome [14, 23]. Both WT and *fad2-1a fad2-1b* mutant soybean lines were transformed; from four independent transformations, a total of 72 events were generated (Table 1). To detect TALEN-induced mutations, the *FAD3A* gene was amplified and digested with T7 endonuclease I. We observed that 16 of the 72 events had cleavage products consistent with mutations within the GmFAD3_T02.1 target sequence. Cloning and sequencing of *FAD3A* amplicons revealed that all 16 plants harbored short deletions within the TALEN spacer sequence, ranging from 4 to 135 bp. Together, these results confirm the successful mutagenesis of *FAD3A* within T0 soybean plants, with a mutagenesis frequency of ~22 %.

**Table 1:** Summary of *FAD3A* mutation frequencies within T0 soybean plants

Experiment	Background	Number of events	Number of T7-positive events	Mutagenesis frequency
Gm183	*fad2-1a fad2-1b*	30	8	27 %
Gm184	Bert	35	5	14 %
Gm205	*fad2-1a fad2-1b*	3	2	67 %
Gm206	Bert	4	1	25 %
Average mutagenesis		72	16	22 %

To confirm TALEN-induced mutations can be stably transmitted to subsequent generations, candidate T1 plants derived from experiment Gm183 were screened for mutations within *FAD3A* by PCR amplification and sequencing of clones. From three different T0 events (Gm183-4, Gm183-5 and Gm183-6), we identified T1 plants harboring heterozygous or homozygous mutations within *FAD3A*, indicating that mutations were stably transmitted to the next generation (Table 2). Further, we assessed T1 plants by PCR for the presence of transgene sequence. Of the 25 T1 plants assayed, 20 were positive for transgene sequence and five were negative (i.e., null segregant for the TALEN transgene). Importantly, two of the five transgene-free T1 plants harbored mutations within *FAD3A*. These two plants were self-pollinated to produce homozygous-mutant, transgene-free *fad2-1a fad2-1b fad3a* soybean plants. Notably, we also identified a single-gene *fad3a* knockout T1 plant from experiment Gm184 (identified as Gm184-3-20) which contains a homozygous −4 bp deletion within *FAD3A*. We failed, however, to identify plants with combinations of *FAD3A* and *FAD3B* mutations, indicating that the frequency of mutagenesis at *FAD3B* was <1.4 % (i.e., less than 1 out of 72 events).

**Table 2:** Genotype of T1 plants from candidate T0 events harboring mutations within *FAD3A*

Parent line (T0)	T1 plant number	*FAD3A* genotype	Presence of transgene
Gm183-4	1	−7 bp/WT	Undetected
	2	−7 bp/-7 bp	+
	3	−7 bp/-7 bp	+
	4	−7 bp/WT	+
Gm183-5	2	−43 bp/WT	+
	3	WT/WT	Undetected
	4	−43 bp/-43 bp	+
	5	−43 bp/-43 bp	Undetected
	7	−43 bp/-43 bp	+
	8	−4 bp/-4 bp	+
	9	−43 bp/-43 bp	+
Gm183-6	1	−4 bp/-4 bp	+
	2	WT/WT	+
	3	WT/WT	+
	4	−4 bp/WT	+
	5	−4 bp/-4 bp	+
	6	WT/WT	Undetected
	7	−4 bp/WT	+
	8	WT/WT	Undetected
	9	WT/WT	+
	10	WT/WT	+
	11	−4 bp/WT	+
	12	−4 bp/WT	+
	13	WT/WT	+
	14	−4 bp/WT	+

### Oil from *fad2-1a fad2-1b fad3a* homozygous mutant soybean seeds contains high oleic, low linoleic and low linolenic acid

**Fig. 3:**
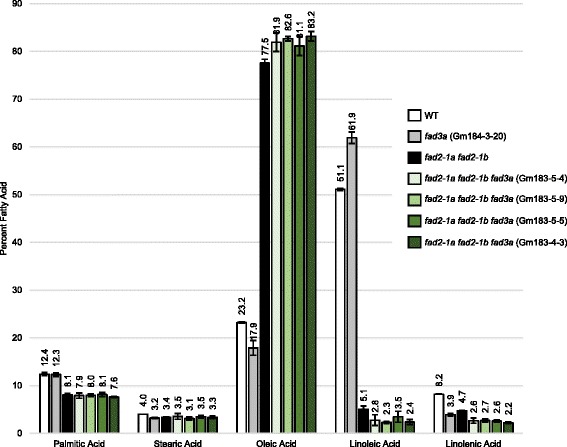
Fatty acid profile from *fad2-1a fad2-1b fad3a* soybean plants. Oil from T2 seed from four different T1 *fad2-1a fad2-1b fad3a* mutant lines was analyzed. The genotypes for the *fad2-1a fad2-1b fad3a* plant lines at the *fad3a* TALEN target site were −7 bp/-7 bp (Gm183-4-3), −43 bp/-43 bp (Gm183-5-4), −43 bp/-43 bp (Gm183-5-5), and −43 bp/-43 bp (Gm183-5-9). The genotype for the *fad3a* plant line was −4 bp/-4 bp (Gm184-3-20). Error bars represent standard deviation of the oil levels within individual seeds, specifically, five seeds for Gm183-4-3, five seeds for Gm183-5-4, five seeds for Gm183-5-5, five seeds for Gm183-5-9, five seeds for Gm184-3-20, four seeds for WT, and 20 seeds for *fad2-1a fad2-1b*

Next, we assessed the oil profile within seed from *fad3a* and *fad2-1a fad2-1b fad3a* homozygous mutant soybean lines (Fig. 3). Seed from T1 homozygous mutant lines were collected and assessed for oil composition by gas chromatographic analysis of fatty acid methyl esters (GC FAME Analysis; Additional file 1). In oil from *fad3a* plants, we observed significant changes in linolenic, linoleic, oleic and stearic acid levels, relative to oil from WT plants. We observed linolenic acid decreased from 8.2 ± 0.4 to 3.9 ± 0.3 %, linoleic acid increased from 51.1 ± 0.2 to 61.9 ± 1.2 %, oleic acid decreased from 23.2 ± 0.8 to 17.9 ± 1.6 % and stearic acid decreased from 4 ± 0.01 to 3.2 ± 0.1 %.

We observed significant changes in fatty acid levels within seed oil from *fad2-1a fad2-1b fad3a* soybean plants, when compared to *fad2-1a fad2-1b* soybean plants. The average linolenic acid level within oil from *fad2-1a fad2-1b fad3a* plants was 2.5 ± 0.4 %, significantly lower than oil from *fad2-1a fad2-1b* soybean plants (4.7 ± 0.1 %). Linoleic acid levels decreased from 5.1 ± 0.7 % in *fad2-1a fad2-1b* lines to 2.7 ± 0.9 % in *fad2-1a fad2-1b fad3a* lines, and oleic acid levels increased from 77.5 ± 0.8 % in *fad2-1a fad2-1b* lines to 82.2 ± 1.6 % in *fad2-1a fad2-1b fad3a* lines. Together, these results indicate that stacking mutations within *FAD2-1* and *FAD3A* genes decreases linolenic and linoleic acid levels to below 3 %, and increases oleic acid levels to over 80 %.

## Discussion

In 2015, the FDA ruled that *trans* fat is no longer ‘generally recognized as safe’ for use in food, and has set a 3 year deadline to remove partially hydrogenated oils from food products. In an effort to improve shelf life and cooking characteristics, soybean oil is partially hydrogenated. However, partial hydrogenation results in increased levels of *trans* fats. Generating soybean oil with lower levels of polyunsaturated fatty acids promises to enhance shelf life and heat stability, thereby reducing the need for hydrogenation. Previously, we generated soybean that produce high oleic acid oil by knocking out both *FAD2-1A* and *FAD2-1B* genes*.* Here, we further improved oil characteristics by decreasing polyunsaturated fatty acids (linoleic and linolenic) to levels below 3 %. The methods and products presented here provide solutions for the demand of soybean oil with increased oxidative stability.

Soybean plants with high oleic acid have been developed by research groups using different approaches. These approaches include RNAi [11], combining naturally occurring or induced mutations [13, 24, 25], and site-directed mutagenesis using sequence-specific nucleases [14]. Of the characterized high oleic soybean lines, most have distinct differences in the levels of individual fatty acids. For example, Monsanto’s Vistive® Gold high oleic acid soybean oil (MON 87705), created using RNAi targeting *FATB* and *FAD2-1* and harboring naturally occurring mutations in *FAD3* genes, has oleic, linoleic, linolenic and saturated fatty acids levels of 71.7, 16.9, 2.9 and 6.8 %, respectively [26]. Further, Pioneer/DuPont’s Plenish® high oleic soybean oil (HOSO 305423), created using RNAi against *FAD2-1*, has oleic, linoleic, linolenic and saturated fatty acids levels of 70.6, 5.5, 7.2 and 14.3 % respectively [26, 27]. The *fad2-1a fad2-1b fad3a* lines created here have oleic, linoleic, linolenic and saturated fatty acids levels of 82.2, 2.7, 2.5, and 11.3 %, respectively. Variations in fatty acid levels may be due to differences in gene targets (e.g., *FATB* vs *FAD3*), genetic background, growth conditions, and possibly incomplete silencing of gene expression when using RNAi technology.

We observed lower levels of linoleic acid and higher levels of oleic acid within oil from *fad2-1a fad2-1b fad3a* plants, when compared to oil from *fad2-1a fad2-1b* plants. It would be expected that knockout mutations within desaturase genes would result in accumulation of the corresponding substrate. Indeed, this is the case for plants containing mutations in either *FAD3A* or *FAD2-1A FAD2-1B*; mutations in *FAD3A* resulted in increased levels of linoleic acid, and mutations in *FAD2-1A FAD2-1B* resulted in increased levels of oleic acid. When we introduced *FAD3A* mutations within *fad2-1a fad2-1b* soybeans, the level of linoleic acid decreased and the levels of oleic acid increased. This trend was also observed in high oleic and low linolenic soybean plants generated after combining different sources of mutant *FAD2-1A, FAD2-1B* and *FAD3A* genes [20]; however, fatty acids levels were significantly affected by environmental conditions. Further, and unexpectedly, we observed that oil within *fad3a* and *fad2-1a fad2-1b* plants had deceased levels of the two fatty acids immediately preceding the substrate of the inactivated desaturase (i.e., palmitic and stearic acid in *fad2-1a fad2-1b* plants, or stearic and oleic acid in *fad3a* plants). Understanding properties of additional soybean desaturase proteins and the effects of genetic background and environmental conditions may provide a better understanding of the lipid biosynthetic pathway in soybean.

Here, we used TALENs to generate *fad2-1a fad2-1b fad3* knockout soybean lines; however, there are other sequence-specific nucleases that can be used for plant genome editing, including meganucleases, zinc-finger nucleases and CRISPR/Cas systems. Three key parameters for choosing a sequence-specific nuclease include efficacy (i.e., how likely will the nuclease introduce a desired modification), target site specificity, and ease of construction. Although meganucleases and zinc-finger nucleases have achieved acceptable mutation frequencies and target site specificity [28, 29], their widespread use has been hindered due to difficulties with construction [30, 31]. TALEN and CRISPR/Cas systems have overcome these challenges as they have a modular ‘one RVD to one base pair’ design or a RNA-DNA interaction, allowing for efficient reconstruction of nucleases with altered target sites. One difference between TALENs and CRISPR/Cas is target site length. CRISPR/Cas9 from *Streptococcus pyogenes* recognizes, in general, 17–20 nucleotides of sequence plus a three nucleotide PAM sequence (NGG), providing ~19-22 nucleotides of target site specificity. TALEN pairs, on the other hand, are frequently engineered to recognize 30–40 nucleotides, which may lead to fewer off-target double-strand breaks. Whereas both TALENs and CRISPR/Cas9 tolerate certain nucleotide changes within their target sequences, both provide sufficient specificity to target a single site within a complex plant genome, provided the target site (or a similar target site) is not repeated elsewhere in the genome.

An advantage of engineering crops with sequence-specific nucleases is that the resulting product is not required to harbor transgenic DNA. Within this study, we identified two modified soybean lines with undetectable levels of transgenic DNA. The genotype of these plants were described to the USDA for the purpose of determining regulatory status. An opinion letter, issued May 20th, 2015, indicated that the resulting *FAD3A* knockout plants are not regulated by the USDA under seven CFR part 340 [32]. This means that trials can be launched with transgene-free *fad3a* knockout plants to assess their phenotype in field grown conditions. Due to the lengthy and costly deregulation process, the technology and methods presented within this study provide a clear advantage over conventional transgenesis, thereby enabling more groups to contribute to crop improvement and food security.

## Conclusions

Here we describe methods to efficiently stack quality traits within plants using sequence-specific nucleases. TALENs targeting FAD3 were directly delivered to soybean *fad2-1a* and *fad2-1b* knockout lines to produce triple knockout *fad2-1a fad2-1b fad3* plants. Seed oil from the triple knockout lines had significantly altered fatty acid levels, compared to the parent *fad2-1a fad2-1b* lines. The polyunsaturated fatty acids, linoleic and linolenic acid, decreased to levels below 3 %, and the monounsaturated fatty acid oleic acid increased to levels over 80 %.

## Methods

### Plant material

Plant material used within this study was from soybean [*Glycine max* (L.) Merr.] variety ‘Bert’.

### Plasmid construction

Coding sequences for the TALEN pairs used in this study (GmFAD3_T01.1, GmFAD3_T02.1, and GmFAD3_T03.1) were synthesized as previously described [33]. Individual TALEN monomers were cloned into protoplast expression vectors harboring a nopaline synthase (NOS) promoter and terminator. TALEN backbone architecture comprised N-terminal truncations (N152: TAAAKFERQHMDSIDIADLRTLGYSQQQQEKIKPKVRSTVAQHHEALVGHGFTHAHIVALSQHPAALGTVAVKYQDMIAALPEATHEAIVGVGKQWSGARALEALLTVAGELRGPPLQLDTGQLLKIAKRGGVTAVEAVHAWRNALTGAPLN) and C-terminal truncations (C40: SIVAQLSRPDPALAALTNDHLVALACLGGRPALDAVKKGL). Each TALEN monomer comprised 15 repeat domains for targeting 15 nucleotides of *FAD3* sequence, as shown in Fig. 1c. Repeat variable diresidues within the TALE repeats included NI (for targeting adenine), HD (for targeting cytosine), NN (for targeting guanine), and NG (for targeting thymine). To facilitate trafficking to plant cell nuclei, an SV40 NLS (PKKKRKV) was added to the N-terminus of the TALEN protein. The size of plasmids encoding TALE monomers was 6151 bp. Plasmids were isolated from bacteria using the QIAGEN® maxiprep kit.

### Soybean transformation

Experiments within this study were performed using the soybean variety, ‘Bert’, and the *fad2-1a fad2-1b* double homozygous mutant soybean line as previously described [14]. Transformation was carried out using following previously described protocols [14, 23]. Briefly, half-seeds were transformed with plasmid sequence encoding TALEN pairs and a selectable marker, and soybean plants were regenerated on medium containing glufosinate [34]. Explants were incubated in a growth incubator at 28 °C with ~110 μmol/m^2^/s of light. Rooted seedlings were transferred to soil containing a peat-based substrate (BM1, Berger, Les Tourbièr Berger Ltee, Saint-Modeste, QC, Canada), and acclimated to ambient humidity.

### Protoplast transformation

TALEN pairs were assessed for activity using soybean protoplasts. Protoplasts were isolated from immature cotyledons similar to previously described protocols [35]. Briefly, immature cotyledons were digested in an enzyme solution containing 0.45 M D-mannitol, 20 mM MES, 2 % cellulose, 0.5 % macerozyme, pH 5.8. Digestion was carried out for 16 h at 25 °C in the dark with shaking at 26 rpm. Protoplasts were passed through a 100 μm cell filter and collected in a 50 mL Falcon tube. Protoplasts were then pelleted by centrifugation at 100 rpm for 5 min. Supernatant was removed and cells were resuspended in WB-N solution (0.45 M D-mannitol, 10 mM calcium chloride, pH 5.8). Protoplasts were transformed using polyethylene glycol 4000 (20 % diluted concentration) for 30 min. For each TALEN pair, ~500 000 protoplasts were transformed with 30 μg of plasmid (15 μg for each TALEN pair). Protoplasts were washed three times in WB-N and transferred to low retention 15 × 10 mm petri plates. Protoplasts were incubated at 25 °C for 48 h before genomic DNA was isolated.

### Genotyping and 454 pyrosequencing

To assess TALEN activity by 454 pyrosequencing, and to determine the genotype of candidate *FAD3* mutant plants, the *FAD3A*, *FAD3B* and/or *FAD3C* TALEN target sequences were individually amplified by PCR. Primers for amplifying the *FAD3A* gene were GmFAD3A_F1 (5’-ACACTGCTTTGTTATGCCTACCTCAT) and GmFAD3A_R1 (5’-CTTCTCGGTTAACTAAGATAATGACAAAAAAAAATG). Primers for amplifying the *FAD3B* gene were GmFAD3B_F1 (5’-TCTCACACATTGTTCTGTTATGTCATTTCTTC) and GmFAD3B_R1 (5’- GTTAACTAAGATAATGACACATAAAAAAGAGCCATG). Primers for amplifying the *FAD3C* gene were GmFAD3C_F1 (5’- GGACATGATTGGTAACTAATTATTATTACAAATTGTTATGTTATGTTATG) and GmFAD3C_R1 (5’-CAAAGATGGGGAAAGGAAGAGTGAATC).

### Oil analysis

Individual T2 seeds from homozygous-mutant *fad2-1a fad2-1b fad3a* T1 plants were isolated and assessed for oil composition, as shown in Fig. 3. Five T2 seeds from each of four different T1 parent plants with a *fad2-1a fad2-1b fad3a* genotype were sampled; 20 seeds from *fad2-1a fad2-1b* lines were sampled, five seeds from one *fad3a* parent was sampled, and four WT seeds were sampled. Seeds were sent to Eurofins BioDiagnostics (507 Highland Drive, River Falls, WI 54022) for fatty acid analysis. Oil composition was determined using gas chromatographic analysis of fatty acid methyl esters (GC FAME Analysis). The fatty acid levels were reported as the percentage of palmitic, stearic, oleic, linoleic, and linolenic acids to the total fatty acids. Raw GC FAME data is presented in Additional file 1.

## Additional file

Additional file 1: GC FAME data contains the raw GC FAME data used to generate the graph in Fig. 3. (XLSX 18 kb)

## Abbreviations

FAD2: Fatty acid desaturase 2
FAD3: Fatty acid desaturase 3
FDA: Food and drug administration
GC FAME: Gas chromatographic analysis of fatty acid methyl esters
GRAS: Generally recognized as safe
NHEJ: Non-homologous end joining
NOS: Nopaline synthase
SNP: Single nucleotide polymorphism
TALEN: Transcription activator-like effector nuclease
